# Differential Temporal Perception Abilities in Parkinson’s Disease Patients Based on Timing Magnitude

**DOI:** 10.1038/s41598-019-55827-y

**Published:** 2019-12-23

**Authors:** Matthew Bernardinis, S. Farokh Atashzar, Mandar S. Jog, Rajni V. Patel

**Affiliations:** 1 School of Biomedical Engineering and Faculty of Engineering, University of Western Ontario (UWO), London, Canada; 2 Department of Clinical Neurological Sciences, University of Western Ontario (UWO), London, Canada; 3 Canadian Surgical Technologies & Advanced Robotics (CSTAR), London, Canada; 4 Movement Disorders Centre, London Health Sciences Centre, London, Canada; 5 Electrical and Computer Engineering, and Mechanical and Aerospace Engineering, New York University (NYU), New York City, United States of America

**Keywords:** Neurophysiology, Parkinson's disease, Parkinson's disease, Neurological disorders

## Abstract

Non-motor symptoms in Parkinson’s Disease (PD) predate motor symptoms and substantially decrease quality of life; however, detection, monitoring, and treatments are unavailable for many of these symptoms. Temporal perception abnormalities in PD are generally attributed to altered Basal Ganglia (BG) function. Present studies are confounded by motor control facilitating movements that are integrated into protocols assessing temporal perception. There is uncertainty regarding the BG’s influence on timing processes of different time scales and how PD therapies affect this perception. In this study, PD patients using Levodopa (n = 25), Deep Brain Stimulation (DBS; n = 6), de novo patients (n = 6), and healthy controls (n = 17) completed a visual temporal perception task in seconds and sub-section timing scales using a computer-generated graphical tool. For all patient groups, there were no impairments seen at the smaller tested magnitudes (using sub-second timing). However, all PD groups displayed significant impairments at the larger tested magnitudes (using interval timing). Neither Levodopa nor DBS therapy led to significant improvements in timing abilities. Levodopa resulted in a strong trend towards impairing timing processes and caused a deterioration in perceptual coherency according to Weber’s Law. It is shown that timing abnormalities in PD occur in the seconds range but do not extend to the sub-second range. Furthermore, observed timing deficits were shown to not be solely caused by motor deficiency. This provides evidence to support internal clock models involving the BG (among other neural regions) in interval timing, and cerebellar control of sub-second timing. This study also revealed significant temporal perception deficits in recently diagnosed PD patients; thus, temporal perception abnormalities might act as an early disease marker, with the graphical tool showing potential for disease monitoring.

## Introduction

Parkinson’s Disease (PD) is a progressive neuro-degenerative disease generally characterized by neuronal death in the basal ganglia (BG), leading to heterogenous motor abnormalities^1,2^. Non-motor symptoms are also present in the vast majority of PD patients throughout all disease stages^3,4^. Although these non-motor symptoms were classically not considered substantial factors of PD, they are increasingly being shown to contribute to decreased patient quality of life, in many cases to a greater degree than motor-symptoms^3–7^. Numerous common PD non-motor symptoms such as olfaction disturbances and rapid eye movement sleep behaviour disorder frequently predate the appearance of motor symptoms^8,9^. Accordingly, extensive work has studied the use of non-motor symptoms as early disease markers; however, this has not yet led to reliable methods for the early detection of PD^10,11^. Although non-motor symptoms are known to be both important factors of PD and in some cases potential disease markers, accurate diagnosis and treatment of these symptoms remains a challenge^12^. Further shortcomings in effective treatment and monitoring of non-motor features arise from gaps in knowledge regarding the extent of these symptoms.

Of the studied non-motor deficits occurring in PD, abnormalities in some perceptual processes have been observed^4^. One of the perceptual abnormalities that has been noted in PD is the disruption of temporal perception and temporal processing^13–17^. However, like many studies analyzing perceptions in PD, past assessments of timing have often required patient movement. As movements are impaired in PD, the timing aspects of these studies are confounded and the main source of the observed deficits is unknown^14,18–20^. It is not clear whether the reported deficits in perceiving the fabric of time affecting PD patients arise due to impaired temporal processing, impairments in motor timing, or both. The current study sought to address these issues by isolating temporal perception from related motor actions allowing for its independent analysis. To do this, a novel computer-generated graphical tool was developed and utilized to quantitatively assess visual temporal discrimination independent of participation movement in patients with PD. As sensory symptoms like olfaction deficits (which arise in up to 90% of PD patients) are noted as potential biomarkers for PD^4^, there is the possibility of other perceptual PD biomarkers. Furthermore, past work has shown that abnormal neural connectivity occurring during attention-demanding temporal perception tasks is a distinguishing factor between PD patients with no clinically significant cognitive impairments and control participants^21^. Thus, temporal perception can potentially be used in the assessment and tracking of PD. Accordingly, a visual computer-based graphical tool was designed to help track and diagnose PD, providing a simple assessment that can be used in any setting.

Further shortcomings of work studying temporal perception in PD are seen with few perceptual discrimination (ability to discern between two stimuli differing in magnitude) studies occurring in favour of detection (ability to detect stimuli apart from a baseline) tasks, with many of these past discrimination studies involving goal-directed movements and one neural timing mechanism (i.e. interval timing in the range of seconds)^21,22^. This is exemplified in research conducted by Artieda *et al*. that concluded that those with PD display deficits in both motor timing tasks and time estimation tasks across multiple sensory modalities^14,19^. Temporal discrimination is used daily in everyday activities as it is involved in processing subjective timing (an individual’s perception of the amount of time that has passed since a certain event)^23,24^. Based on current subjective timing models, different neural regions are responsible for timing operations depending on the timing scale^24,25^. The BG for example is believed to be an important component in the perception of time in the range of seconds to minutes, while little evidence points to its involvement at the sub-second timing range^24^. As PD does not cause dysfunction in all neural regions–but substantial abnormalities in some regions such as the BG–it is possible that timing processes of scales utilizing neural regions heavily affected by PD will be affected, whereas other timing scales will not be heavily affected in PD. For the reasons discussed above, the computer-generated graphical tool was designed to test visual temporal discrimination abilities of PD patients in the range of hundreds of milliseconds to seconds to address these knowledge gaps.

The primary patient group for the study consisted of mid-stage PD patients using Levodopa oral medication (half life: 0.5–1.5 hours) to restore neural dopamine levels. Levodopa’s reported effect on temporal perception of PD patients has confounding results, with some work suggesting Levodopa improves internal clock function of PD patients^19^, and others suggesting its use leads to temporal impairments^26,27^. Levodopa generally produces positive outcomes for the movement abnormalities of PD (such as tremor and bradykinesia), however recent MRI work suggests that it also enhances the weakened connectivity between the cortico-striatal-thalamo-cortical (CSTC) motor circuit that is involved in the control of timing and movement^28,29^. Dopamine’s influence on timing events extends to internal time keeping allowing for accurate and precise time estimations, reproductions, and perceptions, as well as direct modulation of CSTC connections involved in motor timing^18,30–32^. Furthermore, the ideal dosage of dopamine for treating PD motor symptoms contributes to the “migration effect” occurring in PD, in which small time frames are over estimated and large time frames are underestimated^33^. Levodopa in general has a variable effect for non-motor PD symptoms, often eliciting no effect or detrimental effects^5,34^. Due to Levodopa’s confounding outcomes in past work and the inclusion of movements in past PD temporal studies, the effect of Levodopa on visual temporal perception is still not known. An additional two PD patient groups consisting of fewer patients were also tested. One of these groups consisted of early-stage de novo patients (patients who are not yet using any PD therapies^35^). The third patient group that was studied had mid- to late-stage PD and exclusively utilized Deep Brain Stimulation (DBS) of the subthalamic nucleus. The outcome of DBS use is similar to that achieved from Levodopa use, leading to substantial motor improvement^36^. Also like Levodopa, the non-motor effects of DBS therapy are variable and in many cases unknown (such is the case with temporal perception). Considering the above, the effect of Levodopa and DBS therapy on visual temporal perception is still not fully understood, even though this perception is critical for interacting in dynamic environments. Patients who were using a PD therapy were studied ON and OFF their respective treatments to analyze their efficacy regarding temporal perception.

The main unit used to quantify an individual’s perceptual abilities was the difference threshold (DL; minimum magnitude change needed to differentiate a stimulus from a standard stimulus). Two standard stimuli (0.5 seconds and 1 second) were used to test temporal perception for timing magnitudes of sub-seconds (via sub-second timing) and seconds (via interval timing) respectively. The standards chosen as sub-second and interval timing utilize different neural regions and mechanisms^24^, allowing us to analyze perception of PD patients at both of these timing magnitudes. Furthermore, attentional and memory deficits that often affect PD patients would be exacerbated by the testing of greater time magnitudes^37–39^). Longer durations would also substantially increase testing times, further risking invalid data due to patients experiencing increased fatigue leading to attentional slips. In addition to perceptual sensitivity (DL), this study sought to analyze visual temporal perception coherency in PD patients according to Weber’s Law. Work done by Weber and Fechner^40,41^ led to Weber’s Law, stating that a person’s difference threshold (DL) is directly related to the magnitude of the standard stimulus for a given sensory modality. The ratio of DL to a standard stimulus is constant across different magnitudes of stimuli, displayed through Weber’s Fraction (WF; defined as *WF* = *DL*/*S*, where S represents the standard stimulus magnitude). The majority of perceptions analyzed using WF have validated Weber’s Law, with exceptions seen at extremely low stimuli magnitudes^40,41^. The effect of PD on temporal perception coherency has not yet been observed, motivating this study component. Countless past works have shown timing abnormalities to occur in PD, although many involve motor timing or perceptions linked to movements. Furthermore, the BG appears to have a central role in timekeeping (in the seconds to minutes range) that is directly modulated by dopamine^24,42–44^. The BG is theorized to increase the frequency of pulsator pulses that are collected and measured to determine time durations^30,32^. Increased dopamine levels in the CSTC circuit also reduces uncertainties in time estimations^32^. Alterations in BG activity (due in part to dopamine deficiency) appear to cause disruptions in the internal clock’s core timer, motor timing, and decision making involved in time perception^13,22,45,46^. The above mentioned comments lead to our primary hypotheses that (A) *Visual temporal perception (including perceptual coherency) of PD patients is impaired compared to control participants*, and (B) *Levodopa will reduce the visual timing disturbances in PD patients by restoring BG function, thus tightening the boundaries of temporal function*. Analysis of specialized small patient groups were also conducted to see the effect that DBS has on temporal perception, as well as to analyze if visual temporal perception is abnormal in early-stage de novo PD patients. As DBS improves BG function (acting similarly to Levodopa), it is also hypothesized that it will provide benefits to a PD patient’s visual temporal perception. Due to the BG still being impaired to some extent in de novo patients, it is hypothesized that the early stage PD patients will display impairments in visual temporal perception. This paper systematically analyzes visual temporal discrimination at different time scales in PD patients independent of goal directed movements that could influence participant timing ability, while evaluating the effect of the two common PD therapies and analyzing the coherency of the perceptual capability being studied.

## Results

### Demographic data, PD-related clinical characteristics

A total of 37 PD patients were tested: 25 (22 male, 3 female) who use Levodopa, 6 (4 male, 2 female) who use DBS, and 6 (4 male, 2 female) de novo patients not currently using any medication for their PD; as well as 17 control participants (14 female, 3 male). All participants were residing in the Southern Ontario region at the time of testing. Clinical and demographic data related to the PD patients are shown in Table 1. Oculomotor examination was conducted on all patients by an experienced clinician, and only those without deficits were recruited. It should be noted that due to repeated use of the t-test (for comparisons of perceptual abilities) and Pearson correlation (for analysis of perceptual coherency) the Bonfferoni correction (*α*/*T* in which T is the number of tests performed^47^) was applied, changing *α* from 0.05 to 0.005 and 0.00625 for achieving statistical significance in the analysis of perceptual ability and perceptual coherency respectively.

**Table 1:** Demographic and clinical data for PD patients.

Demographic Data	Levodopa	DBS	De Novo	Control
Number	25	6	6	17
Age (years)	70.04 ± 6.80	55.16 ± 8.89	74.17 ± 3.97	67.71 ± 8.82
Gender (m/f)	22/3	4/2	4/2	3/14
Total Years of education	13.4 ± 2.14	13.33 ± 2.50	13.00 ± 1.67	13.76 ± 1.80
Years since diagnosis	6.88 ± 4.36	11.5 ± 4.04	3.12 ± 2.0	N/A
Clinical Data				
MoCA (out of 30)	26.68 ± 2.17	26.67 ± 3.08	27.83 ± 2.14	27.23 ± 1.59
UPDRS motor sub-scale OFF Therapy	23.92 ± 6.69	34 ± 10.51	22.33 ± 7.91	N/A
UPDRS motor sub-scale ON Therapy	14.72 ± 6.07	22.33 ± 7.92	N/A	N/A
UPDRS motor subscale OFF vs ON Difference	9.20 ± 5.09	21 ± 5.62	N/A	N/A

Abbreviations: MoCA - Montreal Cognitive Assessment; UPDRS - Unified Parkinson’s Disease Rating Scale.

### Temporal perception: healthy vs. PD

**Figure 1:**
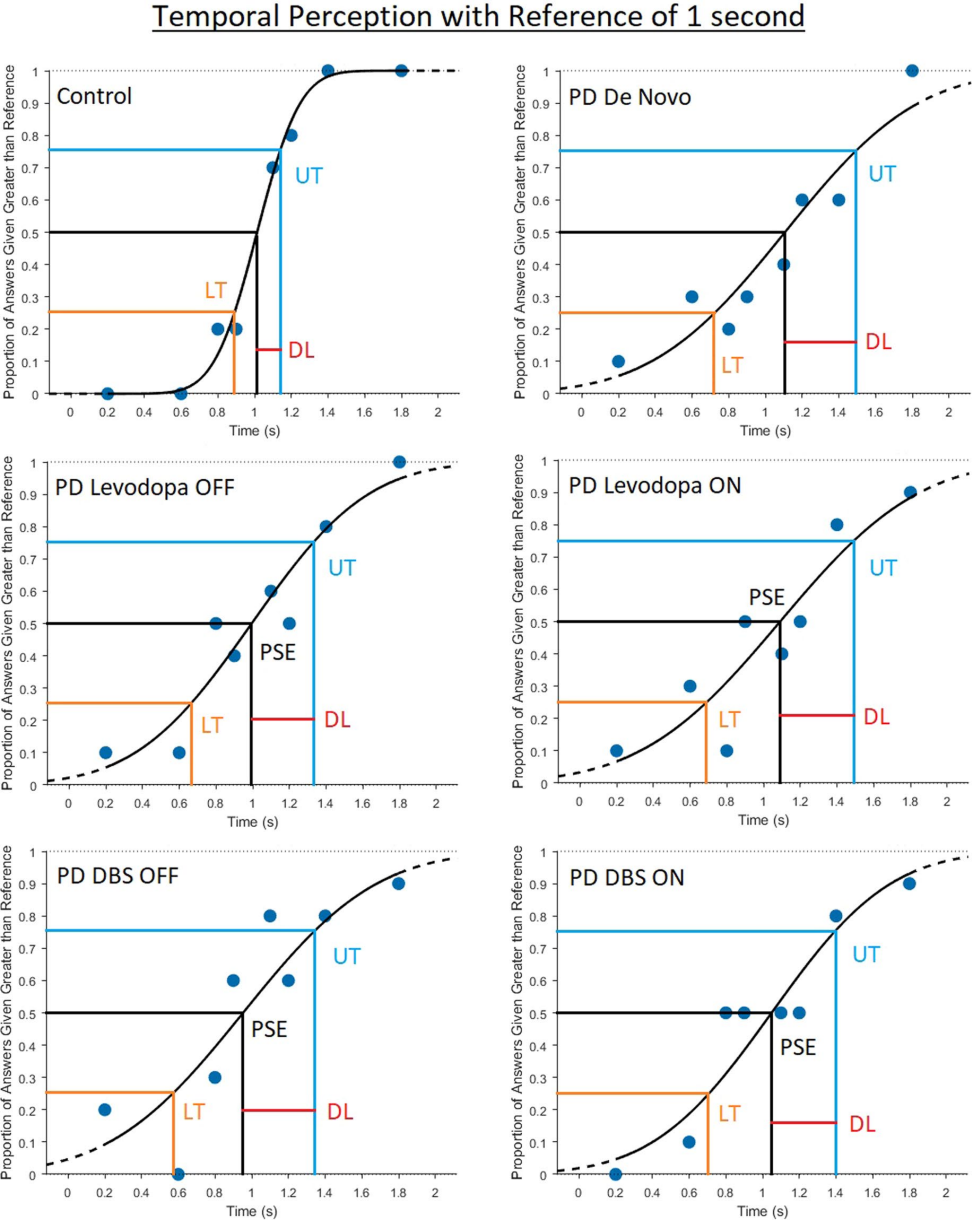
Cumulative Gaussian distribution examples used for subject analysis obtained through assessment of correct and incorrect subject responses given during the visual temporal perception task. Subject DL was analyzed by subtracting the Point of Subjective Equality (PSE) from the Upper Threshold (UT; or subtracting the Lower Threshold [LT] from the PSE). UT and LT are the points of the function which the subject answered correctly 75% of the time for a given standard stimulus. Larger DLs signify decreased perceptual sensitivity.

**Figure 2:**
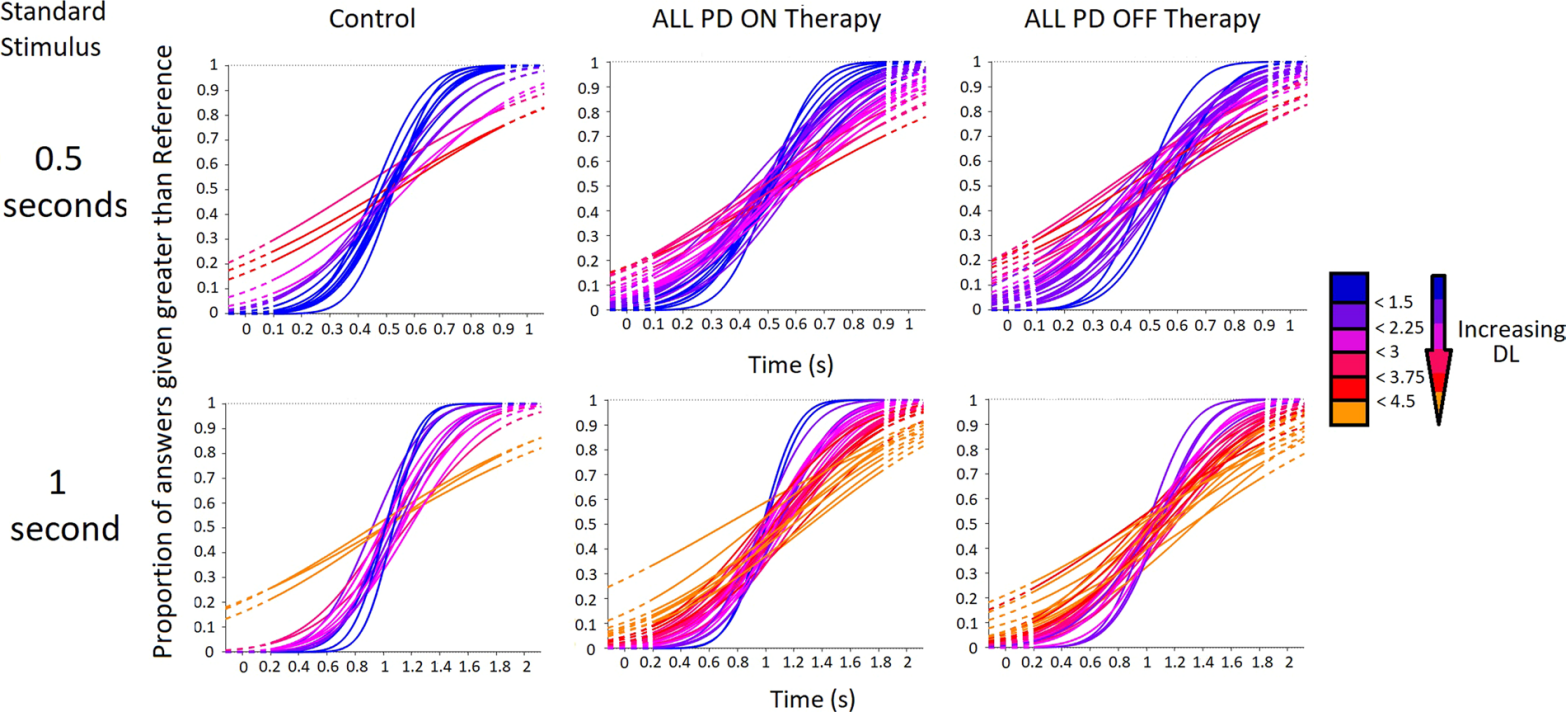
Cumulative Gaussian distributions showing perceptual performance of individual PD participants, categorized by therapy use and standard stimulus. Curves are colour coded based on the participants DL (which is inversely proportional to function slope) for a certain condition. Individual curves that are more blue in colour belong to participants displaying lower DLs (greater slopes) and thus have better perceptual abilities, with red/orange curves signifying the opposite.

DL was used to quantify perceptual abilities, with smaller DLs signifying better perceptual acuity (and thus ability) (Fig. 1). Each participant had two cumulative Gaussian distribution functions produced, one for each of the standard stimuli. The slope of the function is inversely proportional to DL, with steeper slopes indicating smaller DLs, and thus increased perceptual abilities (Fig. 2). The unpaired (independent samples) t-test was used to compare DLs of control and PD participants both ON and OFF Levodopa. Statistical significance was achieved with values of p ≤ 0.005. A datum point was considered an outlier and not considered for statistical evaluation if it was greater than 1.5_X_*Inter*−*QuartileRange*(*IQR*) above the third quartile, or less than 1.5_X_*IQR* below the first quartile.

**Figure 3:**
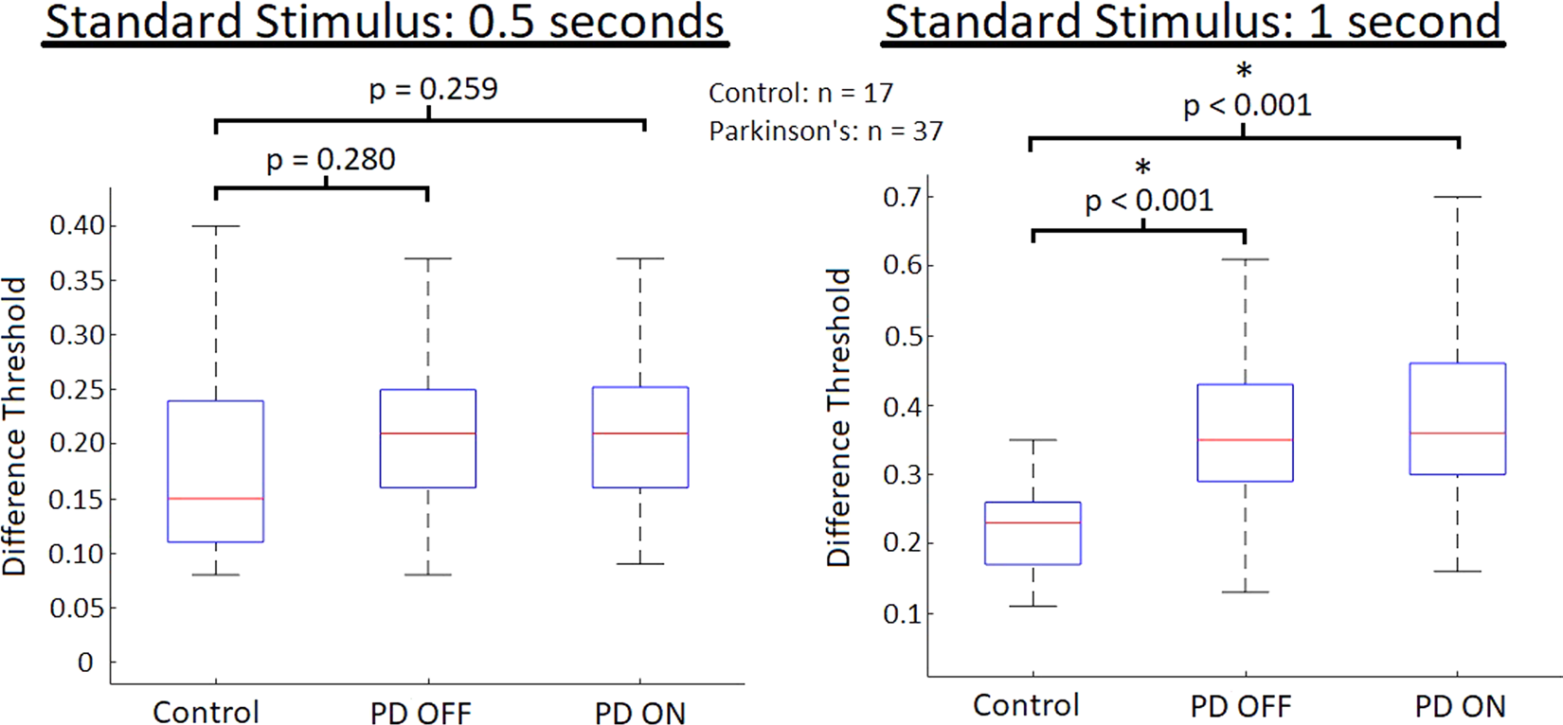
Difference thresholds (DL) inversely proportional to perceptual sensitivity of all PD patients (n = 37) regardless of treatment state compared to control participants. The standard stimulus of 0.5 seconds is displayed on the left, and 1 second on the right; with box-plots related to PD patients OFF and ON their respective therapies. The red lines are the median DL for each group. The bars represent the data spectrum. PD patients did not show any impairments in temporal perception at the standard stimulus of 0.5 seconds ON or OFF PD therapies. However, there were significant impairments seen at the standard stimulus of 1 second OFF PD therapies (p-value < 0.001) and ON PD therapies (p-value < 0.001).

When comparing the DLs for the standard stimulus of 0.5 seconds for all individuals with PD (n = 37) to the control subjects, there were no significant differences in temporal perception between control subjects (average DL: 0.1867 ± 0.1100) and both PD patients OFF their respective therapies (average DL: 0.2150 ± 0.07591; p-value = 0.280; t(51) = −1.092) and ON their therapies (average DL: 0.2181 ± 0.08599; p-value = 0.259; t(52) = −1.140) (Fig. 3). However, when comparing the DLs of control participants for the standard stimulus of 1 second (average DL: 0.2233 ± 0.06773) to all PD patients, the PD patients OFF their respective therapies (average DL: 0.3678 ± 0.1306) had significantly greater DLs (p-value < 0.001; t(46) = −3.909). When PD patients were ON their therapies (average DL: 0.3789 ± 0.1428) they also displayed significant increases (p-value < 0.001; t(46) = −3.883) in DLs for the standard stimulus of 1 second compared to control participants (Fig. 3).

**Figure 4:**
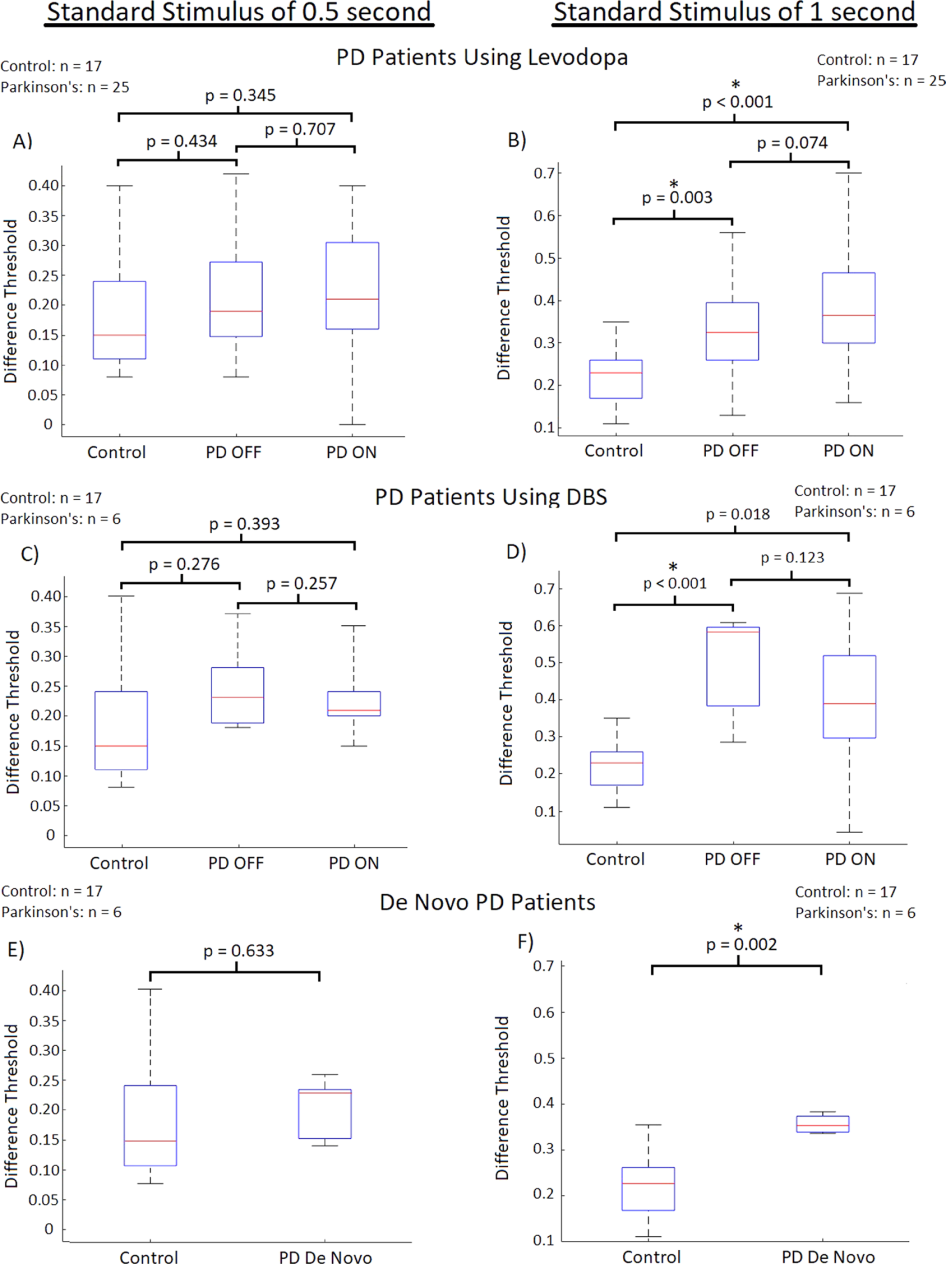
Participant difference threshold (DL; inversely proportional to perceptual sensitivity) separated by individual therapies/de novo obtained through temporal perception examination with the standard stimulus of 0.5 seconds being displayed on the left, and that of 1 second on the right. The red lines are the median DL for each group. The bars represents the data spectrum. Regarding the standard stimulus of 0.5 seconds, there were no significant differences in DLs observed between PD patients OFF and ON Levodopa and DBS compared to controls. De novo PD patients did not display significant differences in DL compared to controls either. Use of Levodopa and DBS therapies did not lead to significant changes in the DLs of PD patients. For the standard stimulus of 1 second, significant differences in DL were observed between PD patients OFF and controls (p-value = 0.003), as well when ON Levodopa and controls (p-value < 0.001) were also seen. PD patients using DBS displayed significant increases in DL when OFF DBS (p-value < 0.001), however when ON DBS no significant DL increases were seen when compared to controls. De novo PD patients also displayed significantly greater DLs than controls (p-value = 0.002) at the standard stimulus of 1 second. No significant differences were seen when PD patients were administered their respective therapies at the larger standard stimulus of 1 second.

As can be seen in Fig. 4A, PD participants using Levodopa as their PD therapy showed insignificant (p-value = 0.434; t(40) = −0.790) differences of DLs (average DL: 0.2101 ± 0.0826) compared to control participants (average DL: 0.1867) when OFF Levodopa at the standard stimulus of 0.5 seconds. When ON Levodopa, PD participants again showed insignificantly (p-value = 0.345; t(40) = −0.956) differing DLs (average DL: 0.2176 ± 0.0979) compared to control participants at the standard stimulus of 0.5 seconds (Fig. 4A). For the standard stimulus of 1 second, PD participants OFF Levodopa displayed significantly greater (p-value = 0.003; t(36) = −3.204) DLs (average DL: 0.3351 ± 0.02436) compared to the control participants (average DL: 0.2233). Again, when using Levodopa, similar results were seen, with PD participants ON Levodopa displaying significantly greater (p-value < 0.001; t(36) = −4.007) DLs (average DL: 0.3806 ± 0.1368) compared to the control participants (Fig. 4B).

It should be noted that both the DBS and de novo PD groups have relatively small n-values (n = 6 each). Thus, the statistical evaluation that was conducted on these groups should serve as observations of interest for these particular groups. The statistics do not necessarily represent these patient populations, however the trends seen do provide insight into the temporal perception abilities of these groups, providing interesting analysis of particular PD patient groups, and rationale to further expand testing to support larger patient groups. Participants with PD who were utilizing DBS therapy in general displayed similar results to PD participants using Levodopa. At the standard stimulus of 0.5 second PD participants OFF DBS displayed insignificant (p-value = 0.276; t(20) = −1.121) DL differences (average DL: 0.2460 ± 0.0760) compared to control participants (average DL: 0.1867). When ON DBS, the PD participants also displayed insignificant (p-value = 0.393; t(21) = −0.872) DL differences (average DL: 0.2290 ± 0.07145) at the 0.5 second standard stimulus compared to controls (average DL: 0.1867) (Fig. 4C). Like participants using Levodopa, at the larger tested standard stimulus magnitude (of 1 second) PD participants OFF DBS displayed significantly greater (p-value < 0.001; t(18) = −6.288) DLs (average DL: 0.5062 ± 0.13661) compared to the control participants (average DL: 0.2233). However, when ON DBS, no significant differences (p-value = 0.018; t(18) = −2.612) were seen between the means of PD participants ON DBS (average DL: 0.3873 ± 0.2182) compared to controls (average DL: 0.2233) at the larger temporal magnitudes of 1 second (Fig. 4D).

The third PD group that was tested consisted of de novo PD patients who were not undergoing any treatment for their PD at the time of testing. At the smaller tested standard stimulus of 0.5 seconds, the de novo PD patients DL did not significantly differ (p-value = 0.633; t(21) = −0.485) in mean DL (average DL: 0.2093 ± 0.04335) compared to control participants (average DL = 0.1867) (Fig. 4E). However, as with all other PD patient groups there was significant increases (p-value = 0.002; t(16) = −3.802) seen in the DLs of de novo PD patients (average DL = 0.35565 ± 0.02146) compared to control participants (average DL: 0.2233) (Fig. 4F).

**Figure 5:**
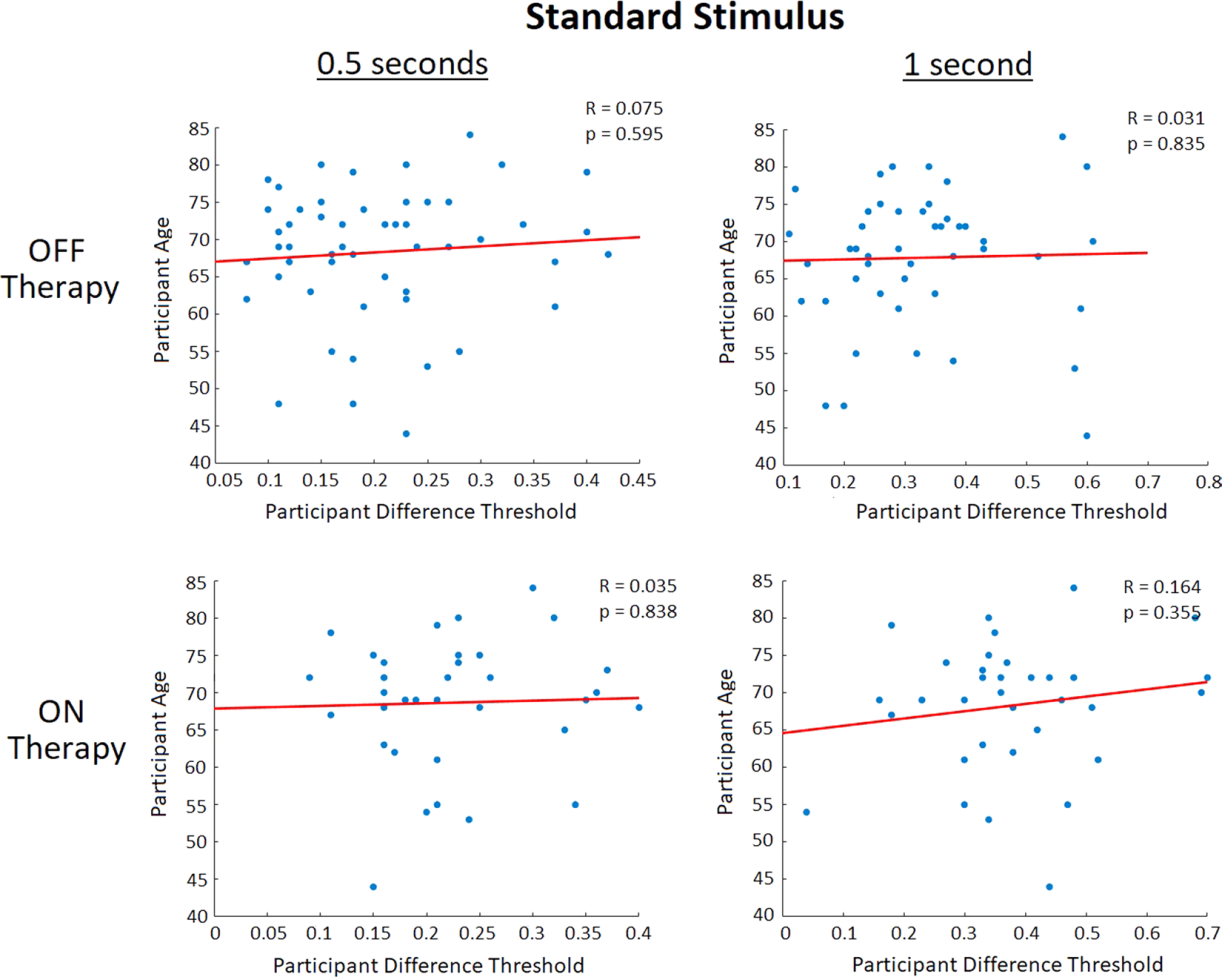
Correlation analysis of all tested study participants comparing an individual participant’s DL (inversely proportional to perceptual sensitivity) to their age. It should be noted that control and de novo participants are included in the OFF therapy figures, but not the ON therapy figures (as they were not using any PD therapies). No correlations were seen between a participant’s age and their perceptual accuracy (as measured via DL) for all conditions (regarding therapy state and standard stimulus).

**Figure 6:**
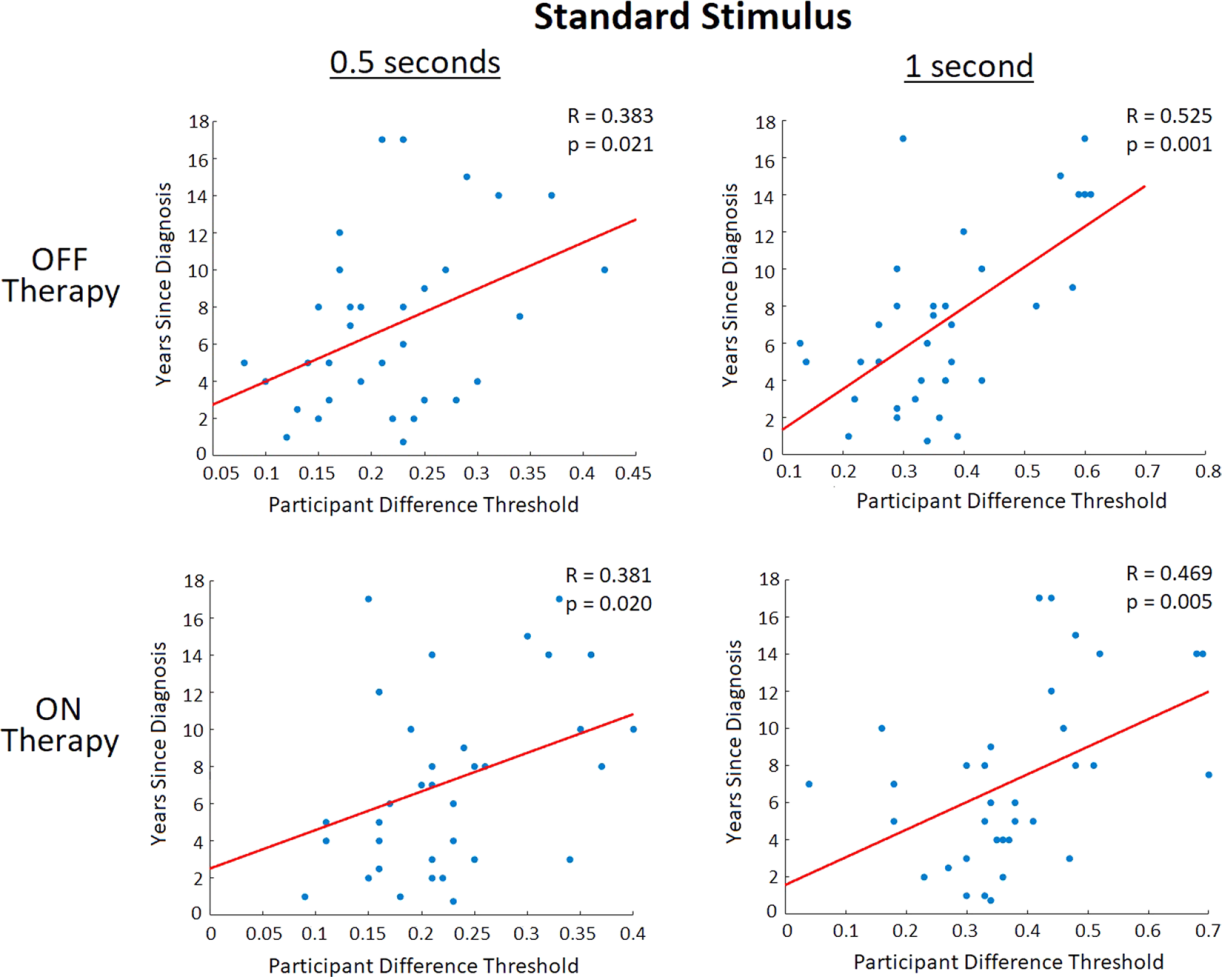
Correlation analysis of all tested PD patients comparing an individual participant’s DL (which is inversely proportional to perceptual sensitivity) to the number of years since they were first diagnosed with PD. For PD patients OFF therapies (including participants using Levodopa or DBS, and de novo participants), there were significant correlations seen between PD duration patient DL at both the 0.5 second standard stimulus (R = 0.383, p = 0.021) and the 1 second standard stimulus (R = 0.525, p = 0.001). Similarly, when patients utilizing PD therapy (Levodopa and DBS) were ON their respective treatments significant correlations between disease duration and DL were seen at both the 0.5 second standard stimulus (R = 0.381, p = 0.020) and 1 second standard stimulus (R = 0.468, p = 0.005).

**Figure 7:**
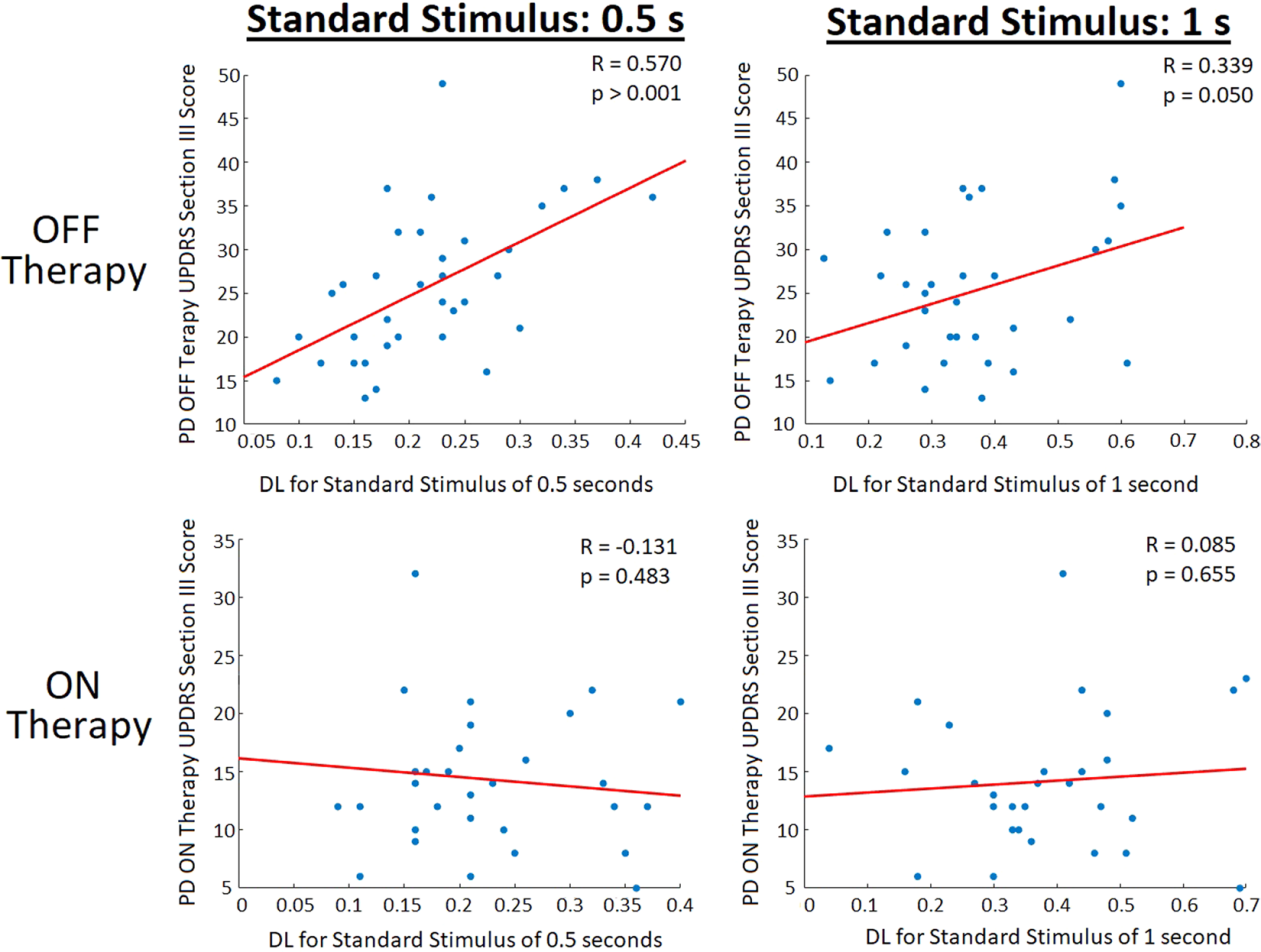
Correlation analysis of all tested PD patients comparing an individual participant’s difference threshold (DL; inversely proportional to perceptual sensitivity) to their UPDRS section III (motor subsection) score. When OFF PD therapies (Levodopa, DBS, and including de novo participants), there were significant correlations seen between the UPDRS section III score and patient DL at both the 0.5 second standard stimulus (R = 0.570, p < 0.001) and the 1 second standard stimulus (R = 0.339, p = 0.050). However, when patients utilizing PD therapy (Levodopa and DBS) were ON their respective treatments no correlations between UPDRS and DL were seen.

**Remark #1**. It should be noted that the average age of DBS patients was significantly lower than that of the Levodopa, de novo and control participant groups (which did not differ between each other). However, age did not appear to affect participants’ DL as no correlations were seen between a participants age and DL (Fig. 5). Furthermore, the duration of PD for DBS patients on average was significantly greater than for the Levodopa and de novo participant groups, which is expected as these patients are at late stages of the disease. Interestingly, when both OFF and ON therapy for the 0.5 and 1 second standard stimuli, significant correlations between a patient’s years since PD diagnosis and DL were seen (Fig. 6). Furthermore, correlations were seen between a PD patient’s DL and their disease induced motor impairment (measured using the clinical standard Unified Parkinson’s Disease Rating Scale (UPDRS) motor subsection diagnostic analysis) when these patients were OFF their respective therapy, with no correlations seen when the patients were using their respective therapy (Fig. 7). While age does not appear to be a contributor to deterioration of the temporal perception ability, disease duration did appear to contribute to an individual’s risk of having developed deficits in temporal discrimination. Movement impairment in PD also appeared to be related to impaired temporal perception, which along with the disease duration results appears to indicate that increased severity of PD also affects visual non-motor temporal perceptions.

### Effect of Levodopa and deep brain stimulation on temporal perception

To analyze the effect that Levodopa and DBS has on the temporal perception of PD participants, the paired two-tailed T-test was used, with statistical significance being achieved with p-values ≤ 0.005. The use of Levodopa did not elicit any significant effects on temporal perception for the PD participants using the therapy. At the smaller tested magnitudes of time using the standard stimulus of 0.5 seconds the use of Levodopa did not significantly alter patient DL compared to when the participants were OFF Levodopa (p-value = 0.707; t(24) = −0.380) (Fig. 4A). At the greater tested magnitudes (using a standard stimulus of 1 second), there were no significant differences (p-value = 0.074; t(23) = −1.873) seen when Levodopa was used, however a potential trend may be present regarding the increase of participant DL when ON Levodopa compared to when OFF (Fig. 4B). Similar to Levodopa, the use of DBS therapy did not elicit significant changes in patient temporal perception. With regard to the smaller standard stimulus (0.5 seconds), the use of DBS did not lead to significant alterations between participants DLs (p-value = 0.257; t(4) = 1.321) (Fig. 4C). At the standard stimulus of 1 second there were no significant differences (p-value = 0.123; t(5) = 1.856) in DL when DBS was turned ON vs. OFF, however again a potential trend regarding the effect of DBS may be present regarding DL improvement when the patients DBS device was turned ON (Fig. 4D). A note of interest, when comparing a participants DL (considering all participants with PD) OFF their respective therapies to the UPDRS subsection III, scores there were significant correlations at both the standard of 0.5 seconds (R = 0.570; p < 0.001) and the 1 second standard (R = 0.339; p = 0.050) (Fig. 7). When PD patients were using their respective therapy however, there were no significant correlations between an individuals DL and UPDRS section III score for both the 0.5 second standard (R = −0.131; p = 0.483) and the 1 second standard (R = 0.085; p = 0.655) (Fig. 7).

### Temporal perception coherency

**Figure 8:**
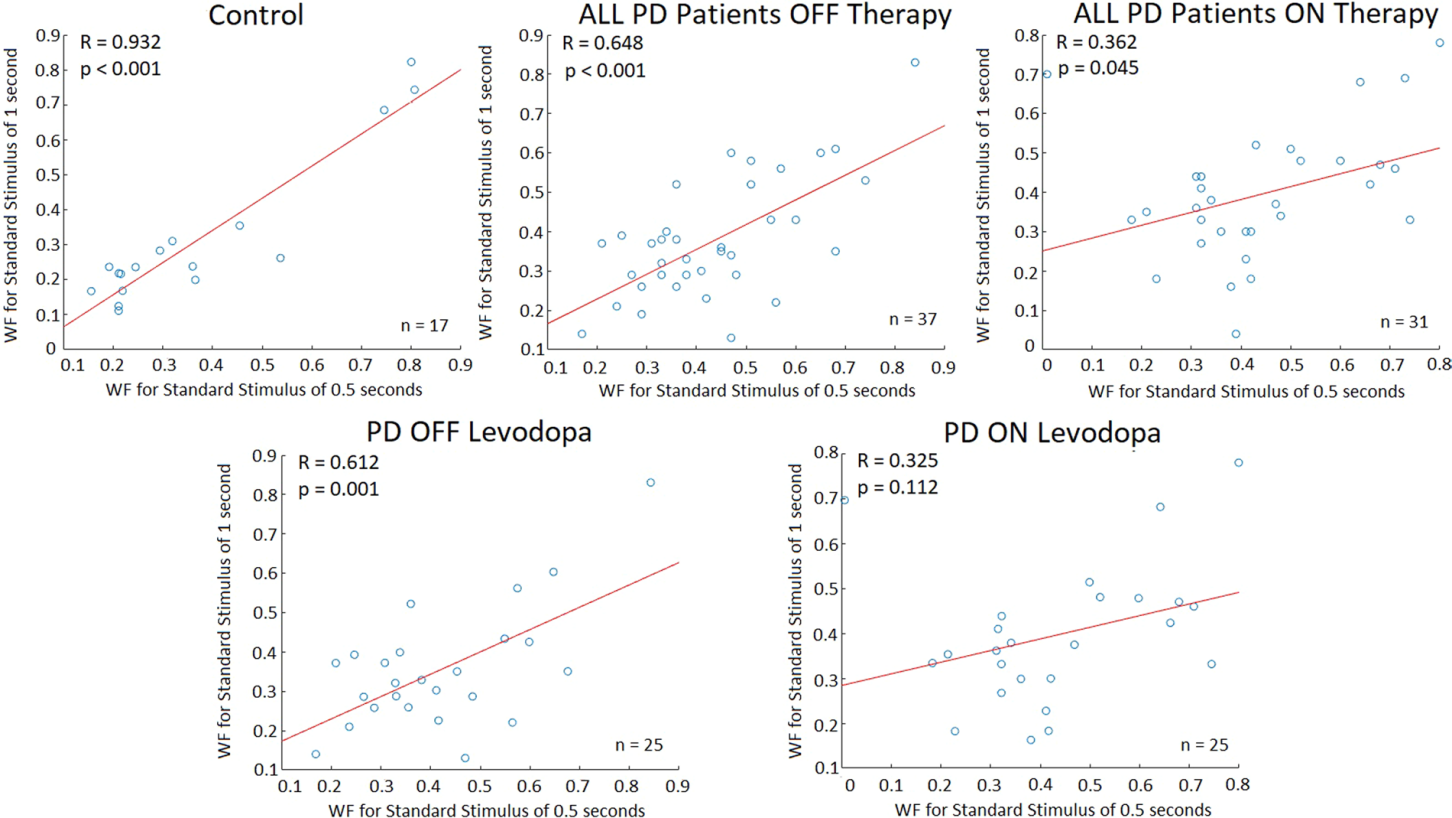
Correlations between participant WF (calculated as the quotient of the difference threshold (inversely related to perceptual sensitivity) and the standard stimulus used to calculate the difference threshold) at the standard stimuli of 0.5 and 1 second. Points displaying high similarity between their x and y values signify that the participant displayed little to no difference in the WF values at different stimulus magnitudes, and thus are in accordance with Weber’s Law. Correlation plots of DBS and de novo patients are not shown due to small sample sizes (n = 6 for each group).

To analyze the perceptual coherency of participants, the WF was calculated at both standard stimuli magnitudes. In normal, healthy conditions, it is expected that there will be strong correlations between the WFs for the different standard stimuli (as an individual’s WF is constant across standard stimuli magnitudes). To analyze this in study participants, Pearson correlation coefficients were applied to compare the similarity between WFs at the two tested standard stimuli. Statistical significance was achieved with values of p ≤ 0.00625. For the control group there were very strong WF correlations between the two tested standard stimuli (Pearson correlation (R): 0.932, p-value < 0.001). Although not as stron a correlation was seen in all PD patients OFF their respective therapies, they still displayed significant, strong correlations between the WF at different standard stimuli magnitudes (R: 0.648, p-value < 0.001). When all PD patients ON their respective therapies were analyzed, no significant correlations were seen between WFs (R: 0.362, p-value = 0.045). When looking at PD patient groups separated by PD therapy usage, PD participants OFF Levodopa also showed strong WF correlations between the two tested standard stimuli (R: 0.612, p-value = 0.001). However, When these PD patients were ON Levodopa they did not display significant correlations between their WFs at the two tested standard stimuli (R: 0.325; p-value = 0.112). For PD participants using DBS, no significant correlations for the WFs between standard stimuli were observed in OFF (R: 0.635, p-value = 0.176) and ON (R: 0.608, p-value = 0.200) DBS states. De novo PD patients did not show any significant correlations between the WFs of the two standard stimuli (R: 0.691, p-value = 0.129) either (Fig. 8).

## Discussion

The current work showed overall that individuals with PD displayed impairments in the tested visual temporal discrimination task (regardless of disease duration) compared to healthy controls. In addition, Levodopa and DBS therapies were shown to elicit minimal effect on the temporal discrimination, and, perceptual coherency was generally disrupted in PD patients. It should be noted that the statistical findings for the DBS and de novo groups should not be considered conclusive evidence for the temporal perception findings in these groups due to their small sample sizes. Instead, these findings provide an interesting view on how patients at different disease stages and utilizing different treatments are affected with regard to temporal perception ability. The main findings of this study are in-line with past work showing impairments in time perception and processing for PD patients, as well as work signifying the BG’s importance in temporal perception at specific time magnitudes^14–17,19,20,48^. The current study however has observed these impairments independent of task-related patient movement, as well as differential perceptual ability based on the scale of time that was used during testing. It should be noted that these impairments are not attributed to abnormalities in oculomotor control, deficits in visual acuity, or clinically diagnosed cognitive deficits common in PD (such as Parkinson’s Disease Dementia [PD-D] or Parkinson’s Disease related Visual Hallucinations [PD-VH]) as participants were tested diagnostically for these symptoms. Furthermore, it is unlikely that the observed deficiencies seen in PD subjects were due to deficits of attention or working memory which commonly affect persons suffering from PD^49^, as there was no deficit seen in the perception of time at the smaller tested magnitudes. Thus, the observed temporal perception impairments are likely due to abnormal timing processes occurring in PD.

Subjective timing processes for different time scales achieve different goals and are controlled by different neural regions^23,24^. In this regard, sub-second timing (processing sub-second time magnitudes) is responsible for proper motor control, speech recognition and production, and playing music^50–52^. The cerebellum has been shown to be the primary neural structure involved in sub-second timing through cerebellar lesion studies of motor timing and rapid, discontinuous timing tasks, with neuroimaging studies providing further evidence^53–56^. Furthermore, based on current research, there is no conclusive evidence that the BG are involved in neural timing in the sub-second range^57^. The current work observed no vision-based temporal perception impairments at the smaller tested standard stimulus (utilizing time scales only in the sub-second range) in PD participants, coinciding with current knowledge regarding subjective sub-second timing. Although connections between the cerebellum and BG exist^58^, there has been no evidence suggesting the BG’s involvement in sub-second timing, which is further confirmed by the results in the current study. Timing in the sub-second range is however important for the control of motor functions and motor timing, which are known to be impaired in PD^20,23,24,59^. These motor timing impairments seen in previous studies can be attributed to abnormal motor function occurring in PD, as suggested by the current study and past work comparing motor timing in PD patients and people with cerebellar lesions^60^. Interestingly, it was previously shown that sub-second timing was heterogeneously impaired in some (but not all) PD patients^61^, however as all tasks involved motor timing these observed abnormalities likely indicate heterogeneous motor timing in the sub-second range for PD patients. The PD patients tested in the current study did show correlations between perceptual impairment and disease duration for both the 0.5 and 1 second standards (Fig. 6). This could indicate that at earlier stages of the disease when the BG is the predominantly affected neural region that timing processes utilizing the BG are impaired. As the disease progresses and spreads to other neural regions, impairments in timing processes that don’t involve the BG might occur (such as sub-second timing). However, this could also be caused by a general increase in impairment (perceptual or otherwise) occurring as PD progresses. The core findings of the current study appear to confirm that timing processes in the scale of hundreds of milliseconds independent of motor functions are not impaired in PD, providing further evidence that the timing control elicited by the cerebellum at this scale functions independently of the BG.

Timing processes in the range of seconds to minutes utilize the interval timing method, which is believed to involve attention of current events and memories of past events to estimate time duration^24^. Interval timing utilizes multiple neural regions including the BG, with the Substantia Nigra Pars Compacta (dopamine producing cells of BG that experience mass neuronal loss during PD) modulating timing processes of the Striatum^24,48,62^. With this considered, past findings of abnormal temporal production, reproduction, and estimation in PD patients aligns with the postulated interval timing models largely involving the BG^14,19,20,59,63^. At the larger tested magnitudes (standard stimulus of 1 second), patients utilizing Levodopa (when both ON and OFF), as well as de novo patients, and DBS patients OFF stimulation displayed significant impairments in temporal perception compared to control participants, with potential trends regarding impaired temporal perception for DBS patients ON therapy also being observed. These findings demonstrate that visual temporal discrimination independent of movement is indeed abnormal in PD, yet there are limitations on these abnormalities based on the BG’s role as an internal clock. Interestingly, observations demonstrating a discrepancy in temporal perception occurring in PD based on the scale of time was seen. This work strengthens postulated subjective timing models in the sub-second range–controlled by the cerebellum with no (or negligible) BG influence–and interval timing models which are largely influenced by the BG. Interestingly, past work investigating weakened CSTC circuit activity in PD that is at least partially responsible for timing deficits suggests increased cerebellar activity occurs to attempt to compensate, potentially indicating entirely different timing processes occur during PD^29^. The current study has also demonstrated that these perceptual abnormalities are occurring early in disease development. As previously studied perceptual deficits occurring in PD such as olfaction often predate disease motor symptoms^4^, it is possible that visual temporal abnormalities also predate motor symptoms, providing an easy to test disease marker.

The use of the two tested therapies did not elicit any significant effects on the visual perception of time. At the lower temporal magnitudes (standard stimulus of 0.5 seconds), this is expected as the postulated timing mechanism is controlled by the cerebellum, which is not dopamine dependent^23,24,27^. The lack of effect caused by Levodopa at the larger tested magnitudes (standard stimulus of 1 second) is more peculiar due to the postulated role of BG in time perception at this scale and the occurrence of reduced striatal dopamine levels in PD^23,24^. Although studies have attributed Levodopa to improved internal clock function^64^, this could be attributed to improvements in working memory (an important component of interval timing) caused by Levodopa. As the time scales were rather small in magnitude for the current study, this may have reduced the errors that occur from abnormal working memory and memory systems rooted in improper striatal activation, which are aided by dopaminergic therapy^22^. Furthermore, the detrimental effect of dopamine antagonists on internal timing seems to be more pronounced than beneficial effects of dopamine agonists^65–67^. Interestingly, if any trend is occurring from Levodopa use it is negative, which has also been observed in previous work^27^. However, based on the statistical analysis, Levodopa did not lead to significant alteration in PD patients’ capabilities in the tested visual perception task. The use of DBS did not lead to significant alterations in a subject’s ability to perform the perceptual task either. However, there was a potential trend towards DBS-based improvements in the perceptual task. Due to the small sample size, it is possible that the statistical analysis is not representative of the effect of DBS on temporal perception; yet, previous work showed neural timing improvements after DBS of the subthalamic nucleus^25^. Based on the finding of the current work, it appears that DBS may be more effective at restoring temporal perceptions in the absence of motor actions, however both PD therapies have minimal if any effect on the tested visual temporal perception. The finding that correlations exist between an individual’s UPDRS part III subsection score and DL OFF the patient’s respective therapy suggest that like motor abnormalities of PD, sensory abnormalities (or at least the tested visual temporal perception) also further deteriorate as the disease progresses. However, the lack of correlations when patients were ON their therapies between a subject’s UPDRS part III score and DL further suggests that while the tested PD therapies do improve movement abnormalities of PD, they do not have the same positive outcome with visual temporal perception (Fig. 7).

**Remark #2**. Of importance, the primary novel findings of this paper highlight the significant effect of PD on the perceptual capability of patients. This study also showed that neither Levodopa nor DBS therapy led to improvements in visual temporal perception. A notable trend however was observed regarding potential impairments in timing processes for the 1 second standard stimulus when Levodopa was used. For the smaller standard stimulus (0.5 seconds) PD patients using Levodopa did not display the same trend (in fact the trend was weakly reversed). None of these trends were statistically significant and no statistical judgement or conclusion has been made for this specific observation. Also, the results of analysis using mixed ANOVA showed that there were no significant relationships between the treatment state (i.e. experiments conducted at different time points) for PD patients at the 0.5 second standard (p = 0.441; F (1, 28) = 0.609) and the 1 second standard (p = 0.215; F (1, 28) = 1.613). Furthermore, there were no observed deficits occurring for DBS patients when ON DBS compared to when OFF DBS (with any potential trends indicating improvement in perception after DBS use). All participants were given at least 1 hour of break time between the ON and OFF states. The above results and notes strongly suggest that there is no potential negative effect on perception caused by repetition in the specific protocol of this work. Further studies, specifically those designed to analyze the effect of repetition can be conducted using the graphical tool presented in this paper to better understand the potential effect of mental fatigue and how it varies between PD and healthy subjects.

Although Weber’s Law is maintained for the majority of tested sensory modalities, past work has questioned its merit in temporal perception^68^. Linear relationships between perceptual accuracy and stimulus magnitude (via Weber’s Law) in temporal perception were shown to occur in healthy subjects between 0.2–2 seconds; however, other work involving visual timing showed consistency in the WF of standard stimuli at 0.6 and 0.9 seconds, but not at 1.2 seconds^68^. In the current work, strong correlations between WFs from the two tested standard stimuli were seen for control participants and PD participants OFF therapy (as well as PD patients using Levodopa when OFF medication). Interestingly, no significant correlations between WF calculations at different standard stimuli were seen when these patients were ON Levodopa. Although perception coherency via Weber’s Law did occur in the tested healthy patients, it is still not certain whether Weber’s Law is maintained in visual temporal perception across a wide range of stimuli magnitudes. However, this finding further promotes the possibility of Levodopa acting negatively in terms of visual temporal perception, prompting further research into both Levodopa’s effect on temporal perception and Weber’s Law in relation to this perception. No correlations in WFs between the 0.5 and 1 second standards were seen in patients using DBS therapy (both ON and OFF) and de novo patients. This is likely partially due to the small sample sizes of each group (*n* = 6 for both groups).

The small sample sizes of both the DBS and de novo groups were a limitation of the current work. Due to restricted patient recruitment/testing time frames, as well as a small candidate pool for the DBS group (with only 10% of potential candidates utilizing the treatment) and de novo group (due to the recruitment centre [University Hospital] being a tertiary care hospital) the recruitment of large numbers of PD patients in these groups was a challenge. However, the results from these groups provide interesting insight into how these populations might function with regards to temporal perception, prompting further research of the groups. Another study limitation was the constant order of patients conducting experiments first OFF PD therapies, followed by ON therapies. This was due to the testing occurring in one day, and the approximate 12 hours needed for an individual to be considered clinically OFF Levodopa (which all non-de novo PD patients were utilizing for treatment). Although this is common practice for PD experiments involving ON/OFF analysis, and extended breaks were provided for participants, fatigue could have occurred for some participants.

The current study demonstrated that abnormalities in visual temporal discrimination independent of goal-directed movement occur in PD in timing scales utilizing interval timing. However, these deficits were not seen with sub-second timing. This supports commonly postulated subjective timing models outlining the BG’s lack of function in neural timing in the sub-second range, and involvement in neural timing in second to minute range. This was also seen in early stage de novo PD patients, suggesting that visual temporal discrimination is disrupted early in the disease. Thus, visual temporal discrimination shows potential as an early disease marker that could be used in diagnostic scenarios, due to the simplicity of testing this perception. A non-invasive, easy-to-use computer graphics-generated tool was implemented to test this perception. The toolbox can be easily modified, allowing for the analysis of different sensory modalities in research or clinical settings. Furthermore, the toolbox is not taxing from a computer processing standpoint, allowing it to be used in a wide variety of clinical and non-clinical settings. Testing of early stage PD patients (specifically those who do not yet display significant motor impairments) should be carried out to further analyze a potential diagnostic use of the toolbox for neurological disorders such as PD. As non-motor PD symptoms often arise before motor symptoms^4^, this tool or similar software could be a valuable asset to assist physicians with early diagnosis of the disease. Furthermore, potential clinical importance of the computer-generated graphical tool are exemplified through its design, as no goal directed movements which could confound perceptual analysis occur, contrasting current diagnostic timing tests (such as the Purdue peg board) which utilize extensive movements^69^. Although movements (via talking) were necessary for the analysis, these actions had zero impact on the analyzed perception as response time was not a considered factor, ensuring motor capabilities had no role in the observations. Due to the simple to use, flexible nature of the graphical tool, its use could assist in the widespread monitoring of neurological disorders, potentially in the comfort of the users home or local community centres should it require further validation. This could allow for more regular disease monitoring that benefits from the tool’s accessibility, further assisting disease prognosis by providing more complete disease analytics for clinicians to utilize. Further studies should also investigate a potential use of the toolbox for predicting the onset of PDD (which may have visual markers such as abnormal colour perception^70^) to further assist physicians with the monitoring of the disease. Many current PD monitoring tools focus on motor symptoms of the disease^71,72^; however, disease phenotype vary from patient to patient, meaning many do not receive optimum treatment for their conditions or realize the extent of their disease symptoms. The graphics-tool used in this study provides a simple means for analysis of non-motor perceptual modalities affected in neurological disorders such as PD. Future work will continue testing visual perceptual modalities in PD, as well as assess perceptual abilities at different disease stages and for different sensory systems (such as auditory perceptions) to further validate the use of perceptual testing toolboxes in clinical and research use. We hope that the computer-generated graphical tool will one day be used in conjunction with other state-of-the-art clinical diagnostic/disease monitoring tools to provide improved clinical outcomes.

## Methods

### Demographics and clinical assessments

**Figure 9:**
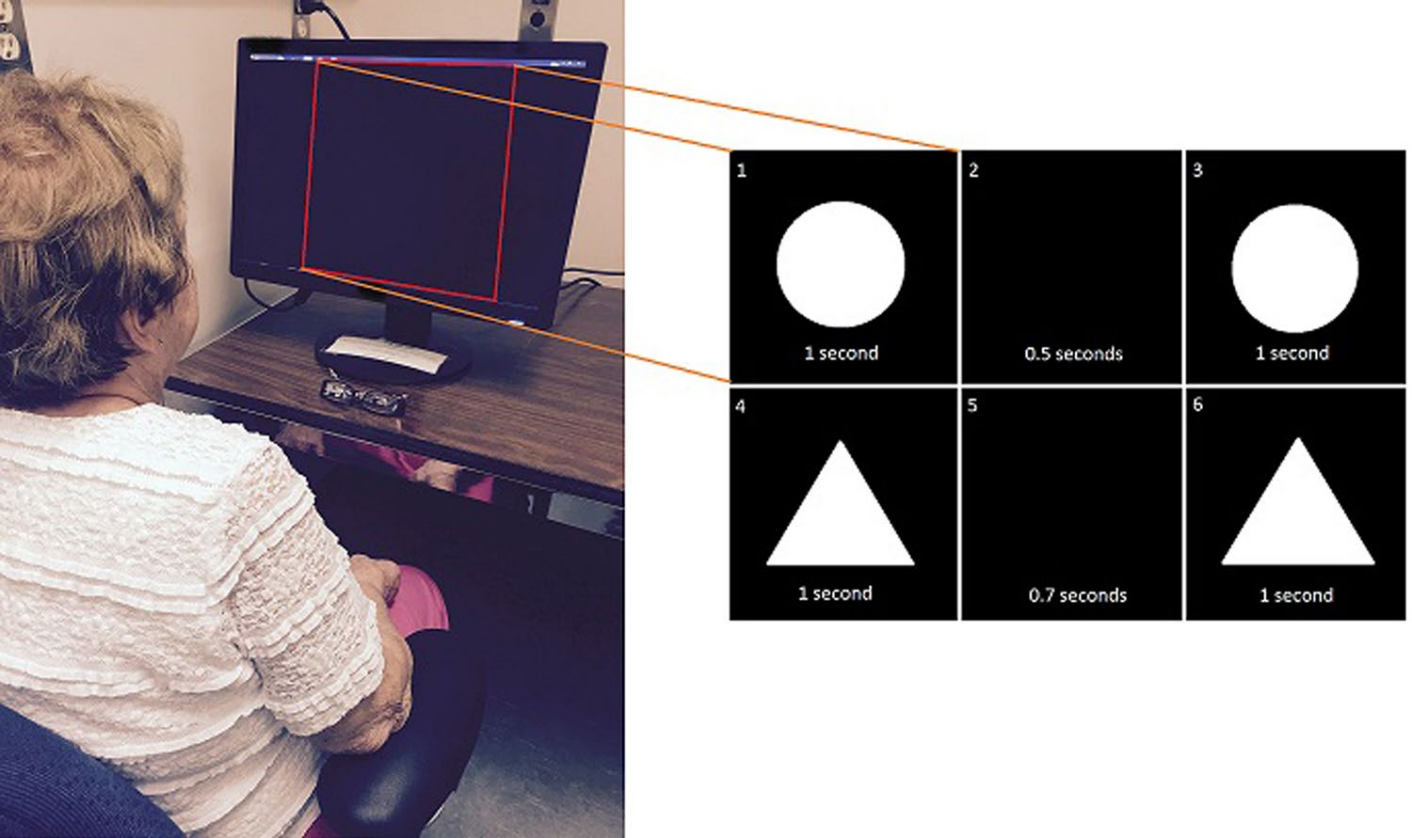
Testing apparatus, with the LG Flatron W2242PM 22 inch (resolution 1680 × 1050) computer monitor at a comfortable viewing position for the participant, who is sitting approximately 2 feet away from the monitor. The testing room has only the participant and experimenter, with excess stimuli (such as sounds, distracting visual) minimized. Illustrative examples of the visual temporal discrimination task shown on right, with each quadrant section representing a specific time window in a single trial on the computer monitor viewed by the participant. In each trial, the participant compares the time period between the appearance of circles to the time period between the appearance of triangles. The participant verbally answers which time period they perceived to be smaller. The example trial begins with Image 1 being shown for 1 second, followed by Image 2 being shown for 0.5 seconds, followed by Image 3 being shown for 1 second. After a 1 second period where the screen is blank, Image 4 is shown for 1 second, followed by Image 5 being shown for 0.7 seconds, followed by Image 6 being shown for 1 second.

The study protocol for this work was approved by the Research Ethics Board of the University of Western Ontario. All experiments were conducted in accordance with the Declaration of Helsinki, as well as the Canadian Tri-Councel Policy Statement of Ethical Conduct for Research Involving Humans. All participants provided informed consent regarding their participation in the study. Furthermore, the participant displayed in Fig. 9 provided consent allowing for their image to be used in publications resulting from the research. All participants in the study were recruited from the Movement Disorders Program at University Hospital, London Health Sciences Centre, Ontario, Canada, where they were diagnosed and have been regularly treated for their PD. Study protocol details and consent forms were provided to patients prior to participation. For this study, 25 participants were recruited who had mid-stage PD (22 male, 3 female) and were on Levodopa therapy. Six patients with mid-late stage PD were recruited who have been receiving DBS therapy (4 male, 2 female), as well as 6 early-stage PD de novo patients (4 male, 2 female) who were not currently receiving any treatment for their PD. Table 1 provides the clinical details and demographic data about the recruited participants. In addition, 17 healthy, age-matched control participants (14 female, 3 male) with no known neurological or psychiatric disorders were recruited for the study. The patients and participants were from the Southern Ontario region of Canada. For this study, all PD patients fulfilled the UK Parkinson’s Disease Society Brain Bank Diagnostic Criteria. Participants utilizing Levodopa therapy refrained from taking the medication 12 hours prior to experimentation, ensuring that they were in the OFF Levodopa state. Similarly, participants receiving DBS had their device turned OFF upon arrival at the testing centre. These participants had to wait at least 45 minutes, ensuring that they were not experiencing any effects from the DBS therapy. After the participants completed the experiment in the OFF state, they were administered 300 mg of Levodopa if this was their primary therapy (unless their regular Levodopa dose was 100 mg or less, in which case they were administered 200 mg); or their DBS device was turned ON to the patients regular stimulation levels if their primary PD treatment was DBS. After an hour’s break the participants went through the experiments again in their ON state. All participants conducted the experiment ON and OFF PD therapy in a one-day testing session. It should be noted that although participants utilizing DBS typically would take it alongside Levodopa, for the duration of the experiment they did not take any Levodopa medication. This was to ensure that the effect of DBS was not confounded by the effect of Levodopa. The severity of motor symptoms affecting the PD participants was assessed using the motor subsection (section 3) of the UPDRS both ON and OFF PD therapy. All PD subjects also conducted a cognitive assessment using the Montreal Cognitive Assessment (MoCA)^73^. Assessments of visual acuity (reading tasks and tests using the Snellen eye chart), smooth pursuit and saccades were carried out for all participants (including control participants). Furthermore, the control group was questioned to detect symptoms of neurological disorder (including PD) as part of the control participant screening. Exclusion criteria (for both control and PD groups) include the presence of considerable cognitive impairments (MoCA < 25), impairments in visual diagnostic tasks, and the presence of visual hallucination (VH). Furthermore, PD patients utilizing pharmacological therapies other than Levodopa were excluded from the study.

### Testing apparatus

A graphics-enabled tool running in Matlab/Simulink was designed at Canadian Surgical Technologies and Advanced Robotics (CSTAR) to examine vision-based temporal perception. The graphical environment of this toolbox can be easily modified, allowing for the examination of various visual sensory modalities. The tool was utilized to study visual temporal discrimination in the current study, with all visual inputs being displayed on an LG Flatron W2242PM 22 inch monitor (resolution: 1680 × 1050). The participants sat approximately 2 feet away from the monitor (Fig. 9). The height of the chair and the monitor were adjusted for optimum comfort. In order to reduce possible visual and auditory distractions, the subjects were located in an isolated room with the experimenter. Figure 9 shows the station utilized for the experiment.

### Experiment

A two-forced alternative choice experiment consisting of 160 trials based on the method of constant stimuli for difference thresholds described by Gescheider^40^ was carried out to examine temporal perception. Each trial in the experiment began with a large, central white circle appearing in the middle of the computer monitor for 1 second before disappearing, leaving a blank screen. After a variable amount of time, the white circle reappears for 1 second, before again disappearing. This is followed by the appearance of a large, central white triangle on the monitor for 1 second, disappearing for a variable amount of time, and reappearing for 1 second before disappearing (Fig. 9). At the end of each trial the subject compares the period of time between the appearance of the circles to the period of time between the appearance of the triangles, verbally answering which time period they perceive to be the shortest (can alternatively be thought of as “which shape was blinking the fastest”). The participant had no time constraints regarding their response, thus although movement was necessary to produce a response, it had no effect on the analysis of patient perception. At the 80th trial, a mandatory break was given to the participants, with as many additional breaks as desired by the participants given throughout the experiment. Two standard stimuli of 0.5 and 1 seconds were tested (where “stimulus” refers to the time duration interval between the two appearences of the same shape), with one of these two standard stimuli being present in every trial. The presentation of stimuli comparisons was completely random, with both standard stimuli being blended together for testing. For each standard stimulus there were 8 comparison values: 0.1, 0.3, 0.4, 0.45, 0.55, 0.6, 0.7 and 0.9 seconds for the standard stimulus of 0.5 seconds; and 0.2, 0.6, 0.8, 0.9, 1.1, 1.2, 1.4 and 1.8 seconds for the standard stimulus of 1 second. The comparison values were chosen so that those differing in magnitude the least from the standard stimulus were answered correctly approximately 50% of the time, and those differing the most in magnitude from the standard stimulus were almost always answered correctly. It should be noted that control and de novo PD patients conducted the experimental task once, where as the PD patients using Levodopa and DBS conducted the task twice (in both their ON and OFF states). It is unlikely that the repetition of the experiment led to improvements based on experiment familiarity however as the PD participants only conducted the task once in a given therapeutic state (with task familiarity not transferring over through therapeutic states). Furthermore, although the enhancement of neural networks related to visual perceptual tasks can occur in adults (i.e. visual perceptual learning), this is a long term change that would not occur in a single experimental session (such as in our work)^74,75^.

### Analysis

The number of correct and incorrect responses were computed for each comparison value of a particular standard stimulus. These values were input into the Psignifit 4.0 third party Matlab toolbox, creating a cumulative Gaussian distribution psychometric function^76^. From the psychometric function the Point of Subjective Equality (PSE), Upper Threshold (UT) and Lower Threshold (LT) (points on cumulative Gaussian distribution function correlating to 0.5, 0.75 and 0.25 points on the x-axis respectively [Fig. 1]) were obtained and utilized to calculate the participants difference threshold (DL), calculated as *DL* = *PSE* − *LT* or *DL* = *UT* − *DL* (Fig. 1). As described by Gescheider^40^, the DL is a value that signifies the difference in the stimulus magnitude necessary for a participant to discern a stimulus as being different from the standard stimulus that it is compared to. Thus, the smaller one’s DL is the more sensitive they are towards the tested perceptual modality at the given standard stimulus^40^. To correct for multiple analyses using the t-test (increasing the risk of type I error), the Bonfferoni correction was used according to the standard formula of *α*/*T*, in which T is the number of tests performed. As 10 t-tests were performed, the corrected *α* for achieving significance was 0.005. Apart from perceptual sensitivity, the perceptual coherency of a participant was also analyzed using Weber’s Fraction (WF). According to Weber’s Law, the ratio of DL to standard stimulus is constant across the magnitudes of stimuli^40^. This is displayed through the WF, defined as *WF* = *DL*/*S*, where S represents the standard stimulus magnitude. To account for the increased risk of a type I error brought on by the multiple correlation analyses (T = 8) the Bonfferoni correction was used adjusting the *α* value needed for significance to 0.00625.

## Data Availability

The data that support the findings of this study are available from the corresponding author upon reasonable request.
